# Impact of respiratory viral infections on mortality and critical illness among hospitalized patients with chronic obstructive pulmonary disease

**DOI:** 10.1111/irv.13050

**Published:** 2022-09-07

**Authors:** Sunita Mulpuru, Melissa K. Andrew, Lingyun Ye, Todd Hatchette, Jason LeBlanc, May El‐Sherif, Donna MacKinnon‐Cameron, Shawn D. Aaron, Gonzalo G. Alvarez, Alan J. Forster, Ardith Ambrose, Shelly A. McNeil, the Serious Outcomes Surveillance and Canadian Immunization Research Network (CIRN) Investigators

**Affiliations:** ^1^ Department of Medicine University of Ottawa Ottawa Ontario Canada; ^2^ Clinical Epidemiology Program Ottawa Hospital Research Institute Ottawa Ontario Canada; ^3^ School of Epidemiology and Public Health University of Ottawa Ottawa Ontario Canada; ^4^ Department of Medicine (Geriatrics), Faculty of Medicine Dalhousie University Halifax Nova Scotia Canada; ^5^ Department of Medicine (Infectious Diseases), Faculty of Medicine Dalhousie University Halifax Nova Scotia Canada; ^6^ Department of Pathology, Faculty of Medicine Dalhousie University Halifax Nova Scotia Canada; ^7^ Canadian Center for Vaccinology (CCfV), IWK Health Center, Nova Scotia Health Halifax Nova Scotia Canada

**Keywords:** COPD, influenza, mortality, respiratory viruses, RSV

## Abstract

**Background:**

Seasonal respiratory viral infections are associated with exacerbations and morbidity among patients with COPD. The real‐world clinical outcomes associated with seasonal viral infections are less well established among hospitalized patients.

**Research Question:**

To estimate the association between seasonal respiratory viral infections, 30‐day mortality, and intensive care unit (ICU) admission among hospitalized COPD patients.

**Study Design and Methods:**

We conducted an analysis of a national prospective multicenter cohort of COPD patients hospitalized with acute respiratory illness during winter seasons (2011–2015) in Canada. Nasopharyngeal swabs were performed on all patients at the onset of hospital admission for diagnosis of viral infection. Primary outcomes were 30‐day mortality and ICU admissions. Secondary outcomes included invasive/non‐invasive ventilation use.

**Results:**

Among 3931 hospitalized patients with COPD, 28.5% (1122/3931) were diagnosed with seasonal respiratory viral infection. Viral infection was associated with increased admission to ICU (OR 1.5, 95% CI 1.2–1.9) and need for mechanical ventilation (OR 1.9, 95% CI 1.4–2.5), but was *not* associated with mortality (OR 1.1, 95% CI 0.8–1.4). Patients with respiratory syncytial virus (RSV) were equally likely to require ICU admission (OR 1.09, 95% CI 0.67–1.78), and more likely to need non‐invasive ventilation (OR 3.1; 95% CI 1.8–5.1) compared to patients with influenza.

**Interpretation:**

Our results suggest COPD patients requiring hospitalization for respiratory symptoms should *routinely* receive viral testing at admission, especially for RSV and influenza, to inform prognosis, clinical management, and infection control practices during winter seasons. Patients with COPD will be an important target population for newly developed RSV therapeutics.

**Clinical Trial Registration:**

ClinicalTrials.gov ID: NCT01517191.

## 1 INTRODUCTION

Respiratory viruses are frequently isolated in nasal and sputum samples from patients experiencing an acute exacerbation of chronic obstructive pulmonary disease (AECOPD).^1^, ^2^, ^3^, ^4^ Previous studies identified the prevalence of respiratory viruses in 30% to 50% of patients with AECOPD.^2^, ^3^ The most frequently identified viruses were rhinoviruses, respiratory syncytial virus (RSV), and influenza, while seasonal coronaviruses, human metapneumovirus (hMPV), adenoviruses, and picornaviruses were isolated less frequently.^2^, ^3^

Influenza infection among hospitalized patients with COPD is associated with high rates of mortality (10%) and critical illness (17%) compared with influenza‐negative patients, but it is unclear if other seasonal respiratory viruses are associated with equally poor prognoses.^5^, ^6^ Studies evaluating the impact of non‐influenza respiratory viruses (NIRVs) among patients with COPD are limited by small sample sizes (<150 patients), examination of limited viral seasons, mixed cohorts of hospitalized and outpatients, and viral testing techniques with variable accuracy.^7^, ^8^, ^9^, ^10^, ^11^ There are no large prospective studies in North America which determine the prevalence and impact of seasonal respiratory viral infections among real‐world hospitalized patients with COPD. Further, prior studies have not robustly assessed the potential differences in morbidity and mortality among viral pathogens.

Understanding the outcomes associated with respiratory viral infection among hospitalized COPD patients is important to guide infection control policy, resource planning, and clinical care; this has been strongly highlighted by the health system consequences of the COVID‐19 pandemic. Understanding the epidemiology of NIRVs is also relevant for development and evaluation of new antiviral therapeutics and vaccines.^12^, ^13^

We aimed to determine the prevalence of respiratory viral pathogens among patients with COPD who were hospitalized with acute respiratory illness during winter seasons, and to study the association between respiratory viral pathogens and 30‐day mortality and intensive care unit (ICU) admissions. This study was conducted over four consecutive winter seasons (2011–2015) in Canada, and therefore, the impact of the COVID‐19 pandemic was not assessed. We hypothesized that respiratory viral infection would be associated with both increased mortality and critical illness among hospitalized patients with COPD, compared with patients without viral infection.

## 2 METHODS

### 2.1 Study design and time frame

We conducted a secondary analysis of a national multicenter prospective cohort study among hospitalized patients with acute respiratory illnesses in Canada over four winter seasons, from November 1st to April 30th annually (2011–2015). Forty‐six hospitals across five provinces participated, representing approximately 10 000 acute care hospital admissions.

### 2.2 Data sources

We used the infrastructure of the Serious Outcomes Surveillance (SOS) network: a national prospective hospital‐based surveillance program to determine and monitor the effectiveness of trivalent influenza vaccination in preventing influenza‐associated hospitalization and mortality among Canadian adults.^14^, ^15^

The SOS network enrolled consecutive adult patients who were hospitalized with acute respiratory illnesses including physician‐diagnosed pneumonia, acute exacerbation of COPD or asthma, unexplained sepsis, respiratory infections, or influenza‐like‐illness symptoms (dyspnea, cough, sore throat, myalgia, fever, or delirium).

Study monitors screened admissions to the ICUs and medical wards to identify eligible study participants and obtained informed consent. All patients underwent testing for influenza at the time of hospital admission with a nasopharyngeal (NP) swab, processed by real‐time reverse transcription polymerase chain reaction (RT‐PCR) or direct fluorescent antibody testing (DFA). Viral testing samples were frozen at −80°C and stored at the Canadian Center for Vaccinology (CCfV) Laboratory in Halifax, Nova Scotia, Canada.

Patient demographics, past medical history, social habits, clinical symptoms, hospital course, length of stay, complications arising during hospitalization, frailty assessments (measured using a previously validated frailty index),^16^ and 30‐day mortality were collected. The SOS study protocol was approved by the research ethics boards of participating institutions.

### 2.3 Inclusion criteria

**FIGURE 1:**
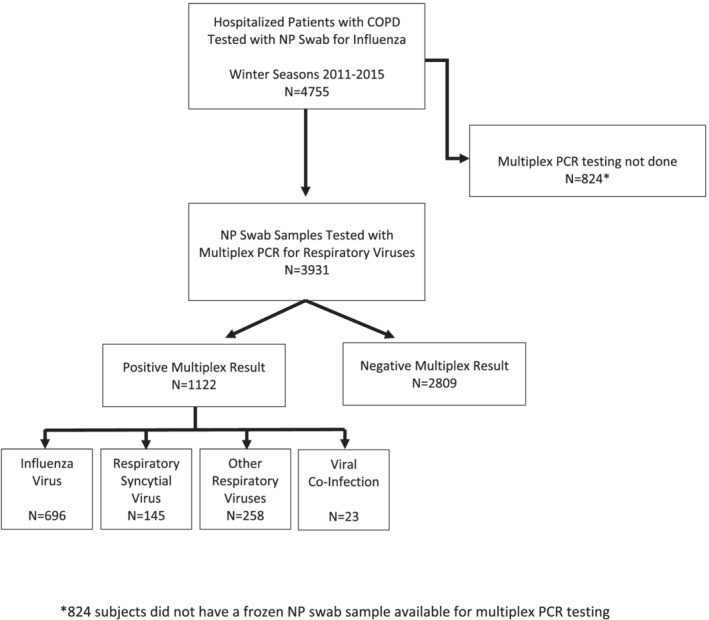
Patients with chronic obstructive pulmonary disease (COPD) who were hospitalized for infectious respiratory symptoms during consecutive winter seasons in Canada, 2011–2015

We included patients in the SOS network who were hospitalized with acute respiratory illnesses, had an NP swab (with subsequent storage at the CCfV laboratory), and a history of COPD (defined by documentation on the medical record). Patients who did not have their NP swab sample stored at the CCfV laboratory were excluded (Figure 1).

### 2.4 Measurements

All NP swab samples were initially tested for influenza virus at the time of hospitalization.^14^, ^15^ Multiplex PCR testing was not routinely available at all sites. Viral samples sent to the CCfV laboratory were thawed from −80°C storage, with 200 μl subjected to a total nucleic acid (TNA) extraction using a MagNAPure LC 2.0 instrument (Roche Diagnostics) with a 100 μl elution, and 10 μl of the TNA was used as template for each of the three multiplex reactions of the Seeplex® RV15 OneStep ACE Detection kit (RV15) (Seegene, Inc., Seoul, Republic of Korea).^17^, ^18^ Quality control testing in the laboratory identified no discordance between RV15 PCR testing on fresh and thawed RSV samples (*data pending publication*).

RV15 multiplex PCR technology can detect multiple respiratory viruses from a single NP swab sample, including influenza A/B, RSV A/B, parainfluenza types 1–4, enterovirus, adenovirus, human metapneumovirus (hMPV), rhinovirus, bocavirus, adenovirus, and seasonal coronaviruses (OC43, 229E/NL63).^19^, ^20^, ^21^ Previous studies using multiplex PCR assays have demonstrated high sensitivity and specificity (90% to 100%) compared with standard culture methods.^18^, ^20^, ^21^

### 2.5 Primary outcomes

Mortality was ascertained by study personnel who contacted the patient or patient designate and reviewed the medical record at 30 days after hospital discharge. ICU admission was defined by admission to an ICU during the index hospitalization.

### 2.6 Secondary outcomes

Secondary outcomes included use of invasive mechanical ventilation or non‐invasive ventilation.

### 2.7 Analysis

Descriptive statistics including means (± standard deviation [SD]), medians (interquartile range [IQR]), and proportions were used to describe baseline characteristics and clinical outcomes among patients with positive and negative NP swab results. Fisher's exact testing was used to assess differences in baseline data and clinical outcomes between patients with positive and negative NP swab results.

Unadjusted primary and secondary clinical outcomes were compared between three groups: individuals with influenza A/B, RSV A/B, and other non‐influenza non‐RSV respiratory viruses. These groups were chosen for comparison a priori due to clinical relevance, as there is currently an approved vaccine and antiviral treatment for influenza, several vaccines for RSV in testing phases, and no widespread adult vaccine or therapeutics available for other NIRVs. Clinical outcomes of mortality and ICU admission have previously been studied among individuals with COPD and influenza infection.^5^

We used multivariable logistic regression to determine the association between molecular identification of respiratory viruses (positive NP swab) and outcomes of 30‐day mortality, ICU admission, and mechanical ventilation, when compared with patients with negative NP swabs.

Subsequent adjusted regression models were used to determine the association between RSV (with influenza infection as the reference group) and 30‐day mortality, ICU admission, mechanical ventilation, and use of non‐invasive ventilation.

All models were adjusted a priori for age, supplemental home oxygen use, and body mass index <18.5 kg/m^2^ (low BMI).^22^ Subsequently, active smoking status, long‐term care dwelling, clinical diagnosis of pneumonia, heart disease, cancer, renal disease, and assisted living dwelling were included in the models and subjected to backwards variable selection.

## 3 RESULTS

### 3.1 Patient cohort

Among 4755 patients admitted to hospital with an acute respiratory illness and comorbid COPD, 3931 (82.7%) had a frozen NP swab available for *testing* with RV15 multiplex PCR (Figure 1). In this cohort, 1122/3931 (28.5%) patients tested positive for a respiratory virus.

### 3.2 Prevalence of respiratory viruses

**FIGURE 2:**
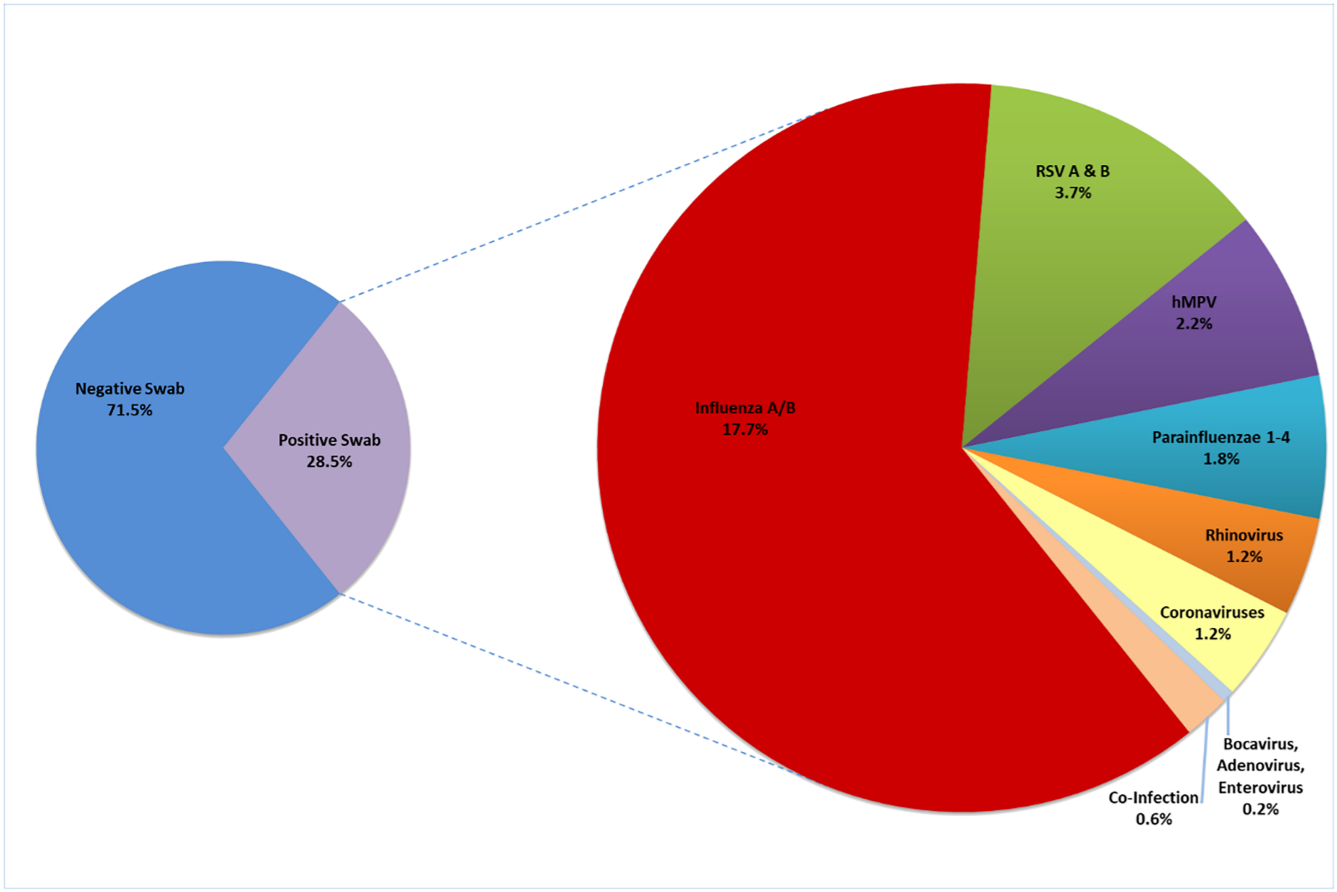
Distribution of respiratory viruses identified by RV15 polymerase chain reaction (PCR) from hospitalized adults with chronic obstructive pulmonary disease (COPD) over four consecutive winter seasons, n = 3931 individuals. *Note*: Seasonal coronaviruses include strains OC43, 229E/NL63. Co‐infections with multiple viruses are detailed in Appendix A.

Figure 2 illustrates the proportion of positive NP swab results and prevalence of individual respiratory viruses isolated by RV15 PCR from hospitalized patients with COPD over four consecutive winter seasons. Influenza A and B were most common (696/3931; 17.7%), followed by RSV A and B (145/3931; 3.7%), hMPV (85/3931; 2.1%), and parainfluenza types 1–4 (71/3931; 1.8%). Seasonal coronaviruses (OC43, 229E/NL63) were less common with 47/3931; 1.2% of total swabs. Rhinovirus, bocavirus, adenovirus, and enteroviruses were found in the remaining 8% of viral isolates. Co‐infection with two viruses was seen in 23/3931 (0.6%) of multiplex PCR samples. Among these co‐infected samples, influenza A/B (16/23) and seasonal coronaviruses (12/23) appeared most frequently (Appendix A).

### 3.3 Characteristics and outcomes among patients with positive and negative viral swabs

Table 1 describes patient characteristics and *unadjusted* clinical outcomes by NP swab result at the time of hospital admission. Patients with positive viral tests were more likely to be living in assisted living or long‐term care facilities (12.5% vs. 10.1% and 8.5% vs. 6.1%, respectively). Baseline degrees of frailty, proportion of patients actively smoking, and cardiac comorbidity were similar between groups.

**TABLE 1:** Baseline characteristics among hospitalized patients with COPD who were tested for respiratory viruses with RV15 multiplex PCR during 2011–2015 winter seasons, n = 3931

Variable	Positive NP swab N = 1122	Negative NP swab N = 2809	p value
Age (mean ± SD) in years	73 ± 12.37	73 ± 11.34	0.86
Age strata (n, %)			
50–64 years	250 (22.3)	592 (21.1)	0.05
65–75 years	319 (28.4)	907 (32.3)	
>75 years	517 (46.1)	1247 (44.3)	
Female sex (n, %)	561 (50)	1372 (48.8)	0.53
BMI kg/m^2^ ≤ 18.5 (n, %)	73 (7.01)	293 (11.1)	<0.001
Active smoking (n, %)	348 (32.8)	825 (29.6)	0.32
Heart disease (n, %)	587 (52.3)	1563 (55.6)	0.06
Kidney disease (n, %)	161 (14.4)	496 (17.7)	0.01
Immunocompromised state (n, %)	249 (22.2)	602 (21.4)	0.61
Malignancy^a^ (n, %)	245 (21.8)	653 (23.3)	0.34
Rheumatic disease (n, %)	74 (6.6)	242 (8.6)	
HIV (n, %)	5 (0.45)	19 (0.68)	
Use of home oxygen (n, %)	150 (13.4)	535 (19.1)	<0.001
Assisted living dwelling prior to admission (n, %)	140 (12.5)	282 (10.1)	0.03
Long‐term care dwelling prior to admission (n, %)	95 (8.5)	170 (6.1)	0.007
Baseline frailty index^b^ (median IQR)	0.22 (0.15–0.3)	0.23 ± (0.16–0.3)	0.37
Unknown (n, %)	362 (32.3)	728 (25.9)	
Influenza vaccination status			
Vaccinated (n, %)	700 (65.8)	1887 (68.9)	0.06
Not vaccinated (n, %)	364 (34.2)	848 (31)	
Unknown (n, %)	58 (5.17)	74 (2.6)	
Clinical diagnosis of pneumonia (n, %)	435 (38.8)	1430 (50.9)	<0.001
Admitting diagnosis of COPD exacerbation (n, %)	584 (52.05)	1306 (46.49)	0.002
30‐day mortality (n, %)	84 (7.5)	222 (7.9)	0.69
Admission to intensive care (n, %)	185 (16.5)	350 (12.5)	0.001
Mechanically ventilated (n, %)	105 (9.4)	158 (5.7)	<0.001
Use of non‐invasive ventilation (n, %)	135 (14.1)	322 (12.9)	0.34

Abbreviations: BMI, body mass index; COPD, chronic obstructive pulmonary disease; HIV, human immunodeficiency virus; IQR, interquartile range; NP, nasopharyngeal; PCR, polymerase chain reaction; SD, standard deviation.

^a^ Malignancy refers to cancer that was treated in the previous 5 years (n = 1 missing value).

^b^ Frailty index is the number of health deficits divided by the total number of deficits measures. It is a score between 0 and 1, where 0.2 represents a frail individual, and higher scores represent increasing degrees of frailty.^14^

*Unadjusted* 30‐day mortality was similar between groups with positive and negative NP swabs (7.5% vs. 7.0%, p = 0.69), while there was more admission to the ICU and need for mechanical ventilation among patients with positive swabs (16.5% vs. 12.5%, p = 0.001 and 9.4% vs. 5.7%, p < 0.001, respectively).

### 3.4 Clinical outcomes among patients with COPD, influenza, RSV, and other respiratory viral infections

Table 2 describes *unadjusted* clinical outcomes among hospitalized patients with COPD with positive NP swabs for influenza A/B, RSV, and other non‐influenza, non‐RSV respiratory viruses.

**TABLE 2:** Unadjusted clinical outcomes among hospitalized patients with COPD who tested positive for influenza, RSV, and other respiratory viruses during winter seasons, 2011–2015, n = 1099

	Influenza A/B	RSV A/B	Non‐Influenza, non‐RSV Respiratory viruses	p value
	N = 696	N = 145	N = 258	
Primary outcomes (n, %)				
30‐day mortality (n, %)	59 (8.5)	4 (2.8)	17 (6.6)	0.04
Admission to intensive care (n, %)	124 (17.8)	26 (17.9)	33 (12.8)	0.15
Secondary outcomes (n, %)				
Mechanically ventilated (n, %)	79 (11.4)	13 (9.0)	12 (4.7)	0.004
Use of non‐invasive Ventilation^a^ (n, %)	67 (11.2)	30 (23.6)	36 (16.8)	0.001

*Note*: Twenty‐three patients who were co‐infected with multiple respiratory viruses were excluded to avoid double‐counting.

Abbreviation: RSV, respiratory syncytial virus.

^a^ Non‐invasive ventilation outcome: 158/1099 (14.4%) patients were missing outcome data.

Unadjusted 30‐day mortality rates were highest among patients infected with influenza (8.5%), and lowest among RSV‐infected individuals (2.8%), p = 0.04. ICU admission rates were equal among those infected with influenza and RSV (17.8% vs. 17.9%), p = 0.15.

Use of mechanical ventilation was highest among the influenza infected group compared with others (p = 0.004), while use of non‐invasive ventilation was highest among RSV‐infected individuals (p = 0.001).

### 3.5 Adjusted association between respiratory viral infection and death, ICU admission, and mechanical/non‐invasive ventilation

Respiratory viral infection (positive NP swab) was *not* associated with significant increase in mortality (OR 1.1, 95% CI 0.8–1.4) after adjustment for age, low BMI, home oxygen use, heart disease, cancer, renal disease, and admission from a long‐term care dwelling. However, viral infection was associated with ICU admission (OR 1.5, 95% CI 1.2–1.9), and with mechanical ventilation (OR 1.9, 95% CI 1.4–2.5) in adjusted models (ICU model adjusted for age, low BMI, home oxygen use, renal disease, active smoking, and clinical diagnosis of pneumonia; Mechanical ventilation model adjusted for age, low BMI, home oxygen use, admission from assisted living dwelling, active smoking, and clinical diagnosis of pneumonia).

Table 3 describes the association between RSV infection and mortality and ICU admission (compared with influenza infection). After adjustment for age, low BMI, use of supplemental home oxygen, and admission from long‐term care, hospitalized patients with COPD and molecular identification of RSV were *less likely* to die (OR 0.24; 95% CI 0.07–0.81), but *equally likely* to require ICU admission (OR 1.09; 95% CI 0.67–1.78) compared with individuals infected with influenza. Other non‐influenza and non‐RSV viral infections (including seasonal coronaviruses) were *less* likely to result in ICU admission (OR 0.64; 95% CI 0.41–0.98).

**TABLE 3:** Adjusted association between respiratory viral pathogens (vs. influenza) and mortality and ICU admission among hospitalized patients with COPD and a positive RV15 multiplex PCR test during winter seasons 2011–2015

Predictor	Odds ratio for 30‐day mortality (95% CI)	Odds ratio for ICU admission (95% CI)
RSV infection vs. influenza infection	0.24 (0.07–0.81)	1.09 (0.67–1.78)
Non‐influenza, non‐RSV, viral infection vs. influenza infection	0.90 (0.49–1.64)	0.64 (0.41–0.98)
Age	1.06 (1.03–1.08)	0.98 (0.97–0.99)
BMI ≤ 18.5 kg/m^2^	1.79 (0.79–4.08)	1.02 (0.51–2.03)
Use of home oxygen	3.32 (1.86–5.93)	1.67 (1.07–2.62)
Admission from long‐term care dwelling	2.66 (1.38–5.14)	
Clinical diagnosis of community acquired pneumonia		1.78 (1.27–2.49)
Current tobacco smoking		1.53 (1.05–2.23)

*Note*: The reference variable is influenza infection (for RSV and other non‐influenza, non‐RSV respiratory viral infections). Variables including age, BMI, use of home oxygen, and admission from long‐term care were forced into the model for mortality because of clinical significance. Clinical diagnosis of community acquired pneumonia and current tobacco smoking status were tested in the model and removed by backwards selection methods due to non‐significance. Age, BMI, and use of home oxygen were forced into the ICU model because of clinical significance. Clinical diagnosis of community acquired pneumonia (CAP), current smoking status, and admission from long‐term care were tested in the model, with CAP and smoking status remaining in the model after backwards selection methods.

Abbreviations: BMI, body mass index; CI, confidence interval; ICU, intensive care unit; RSV, respiratory syncytial virus.

Hospitalized patients with RSV and those with other NIRVs were more likely to require non‐invasive ventilation (RSV OR 3.05; 95% CI 1.82–5.11) compared with patients with influenza in adjusted analyses (Appendix B).

## 4 DISCUSSION

Our analysis of a national multicenter prospective cohort of hospitalized COPD patients over four consecutive winter seasons produced *four* important findings; *first*, seasonal respiratory viral infections are common in this population during winter seasons, and individuals with COPD who reside in long‐term care or assisted living facilities were more likely to test positive at hospital admission. *Second*, RSV and hMPV are the most frequent respiratory viruses identified during winter seasons, after influenza. *Third*, after accounting for important confounding factors, hospitalized patients with COPD and respiratory viral infections have 50% greater odds of ICU admission and 90% greater odds of needing mechanical ventilation compared with those with negative viral tests. *Finally*, RSV infection is associated with significant morbidity among COPD patients (when compared with individuals with influenza infection), with increased ICU admission and need for non‐invasive ventilation.

While influenza is the most prevalent virus (63% of all patients with a positive multiplex PCR test), RSV accounts for the largest portion of non‐influenza respiratory viral infections among hospitalized patients with COPD (145/1099 positive multiplex PCR tests, 13.2%), followed by hMPV (85/1099, 7.7%). Our findings echo those from a small retrospective study among 379 patients with COPD, where the rate of symptomatic RSV illness was 11% and half of those patients required medical attention.^23^ A systematic review of eight observational studies (747 patients) also identified a high mean weighted prevalence of respiratory viruses using PCR techniques (34.1%), although non‐hospitalized patients with COPD exacerbation were also included in the analysis.^3^ This review identified picornaviruses (rhinovirus and enterovirus) to be most common (17.4%), followed by influenza (7.1%) and RSV (5.3%).^3^ Our study, with a fivefold greater number of hospitalized patients, found significantly higher rates of influenza (63% vs. 7%) and RSV infection (13% vs. 5%), and did *not* identify rhinoviruses in significant proportions. It is possible the differences in our findings are due to a temporal effect, as the systematic review included studies conducted between 2001 and 2008, whereas our study was conducted from 2011–2015. Further, the systematic review included non‐hospitalized patients, while our study included *only* hospitalized patients with COPD. Finally, prior observational studies did not account for seasonal biases which can affect viral prevalence.^24^, ^25^ For example, human rhinoviruses are most prevalent during the autumn and spring seasons, while our study was conducted during winter seasons only.^26^

Individuals with a positive viral test at the time of hospitalization were more likely to be residing in assisted living or long‐term care homes, likely reflecting increased risk of virus exposure and host susceptibility (frail individuals). This has significant implications for infection control practice and policy among care homes for vulnerable individuals. A recent survey performed *prior* to the COVID‐19 pandemic assessed infection prevention practices among personnel in long‐term care and found that only 46% of staff surveyed could describe the correct use of infection control precautions, and 42% of respondents reported working while ill.^27^ Further, 54% of staff reported that staying home while ill was difficult due to institutional policy.^27^ Addressing infection control policy and practice in care homes will likely have a significant impact on the burden of hospitalizations and morbidity among vulnerable individuals with chronic lung disease. The current COVID‐19 pandemic has underscored this issue as novel coronavirus infections among staff are associated with deaths among care home residents.^28^

While influenza infections are highly prevalent and associated with death and critical illness, hospitalized patients with COPD and RSV infection also experience morbidity; 24% of patients with RSV infection required *non‐invasive* ventilation (vs. 11% with influenza), and 18% required ICU admission. Once in the ICU, half of patients with RSV required mechanical ventilation. The burden and impact of RSV among hospitalized patients with COPD is not well established in robust prospective studies, although our findings are supported by smaller studies of RSV‐related outcomes in elderly individuals.^29^, ^30^, ^31^, ^32^ We observed a lower mortality rate (4/145, 2.8%) among RSV positive compared with influenza positive individuals. However, a significantly larger proportion of RSV‐infected patients received non‐invasive ventilation (23.6% with RSV vs. 11.2% with influenza), which may have influenced the likelihood of survival in the context of underlying COPD.

To our knowledge this is the largest prospective cohort study (>3000 patients) demonstrating the prevalence and impact of non‐influenza respiratory viral infections among hospitalized patients with COPD. Data were collected over multiple consecutive winter seasons which limits biases introduced by differences in seasonal viral prevalence and strain virulence. We used an active case finding strategy whereby each enrolled patient meeting broadly defined inclusion criteria received a viral swab upon hospital admission.^14^, ^15^ This limits underdetection and biases introduced by analyzing patients who were swabbed for clinical reasons only (i.e., confounding by indication, where patients who are more ill receive viral testing but are also more likely to test positive and have poor clinical outcomes). An active case finding strategy also provides a real‐world assessment of respiratory viral prevalence in this population. Finally, we harnessed the infrastructure of the CCfV to test thawed nasal swab samples with multiplex PCR technology (gold standard) to identify non‐influenza respiratory viral pathogens.

Our study has several limitations. The diagnosis of COPD was ascertained from the medical record, and not by spirometry data. COPD is likely underdiagnosed at the population level and enrolling patients with COPD documented on the medical record likely underrepresents rather than over‐represents this population.^33^, ^34^ We did not have access to lung function data, however supplemental oxygen use and low BMI were use as clinical surrogates for severe lung disease.^22^, ^35^ Although several winter seasons were included, it is possible that some viruses were underrepresented during our study periods if they circulated during the non‐winter seasons (such as rhinoviruses). A recent study suggests that the timing and duration of influenza and RSV epidemics may differ, with influenza epidemics occurring 0–3 months later than RSV epidemics in temperate regions.^36^ According to the Public Health Agency of Canada's surveillance data, the majority of common respiratory viruses are most prevalent during the winter season; therefore, it is unlikely we missed a *significant* portion of viral isolates.^37^, ^38^ We did not have widespread sputum samples for bacteria available in the cohort; however, we adjusted our analyses for a clinical diagnosis of pneumonia, to account for the effect of a concurrent bacterial infection. Lastly, frozen NP swabs were not available for multiplex PCR testing from 824 patients (17%) in our cohort of hospitalized patients with COPD. We could not include these patients in the analysis and therefore the potential impact of a selection bias remains unknown.

In conclusion, seasonal respiratory viral infections among hospitalized patients with COPD are associated with a 50% increase in odds of ICU admission and 90% increase in odds of mechanical ventilation. Our findings suggest that RSV is an important pathogen in this vulnerable population and supports PCR testing for *both* influenza and RSV infection in all patients with COPD who are hospitalized for respiratory illness during winter seasons to inform clinical care, prognostication, infection control practice, and treatment. New and ongoing RSV antiviral treatment and vaccine studies^12^, ^13^, ^39^, ^40^ should include COPD patients to determine whether anti‐RSV therapeutics can reduce exacerbation frequency, hospitalization, morbidity, and mortality.

## CONFLICT OF INTEREST

SM, LY, JL, DM, ME, SDA, GGA, AJF, and AA have no conflicts of interest related to this work to disclose. TH reports grants from GSK and grants from Pfizer, during the conduct of the study. MKA reports grants from Canadian Institutes of Health Research, grants from Public Health Agency of Canada, and grants from GSK, during the conduct of the study and grants from Sanofi, grants from GSK, grants from Pfizer, grants from Canadian Frailty Network, personal fees from Sanofi, personal fees from Pfizer, and personal fees from Immunovaccine Technologies, outside the submitted work. SAM reports grant and clinical trials funding from GSK, Merck, Pfizer and Sanofi, and payments from GSK, Pfizer, Sanofi, and Merck outside the submitted work.

## AUTHOR CONTRIBUTIONS

SM, SAM, MKA, TH, JL, LY, AA, and SDA conceived the study design. SAM, MKA, LY, DM, and ME had direct access to the SOS network data. TH, JL, DK, and ME directed and supervised the processing of multiplex PCR samples. LY and SM conducted and reviewed the initial data analysis. SM drafted the initial manuscript; all authors critically reviewed the analysis and final manuscript and agreed to submit the manuscript for publication.

## GUARANTOR

SM, SAM, and MKA are responsible for the content of the manuscript.

### PEER REVIEW

The peer review history for this article is available at https://publons.com/publon/10.1111/irv.13050.

## Data Availability

The data that support the findings of this study are available from the corresponding author upon reasonable request.

## APPENDIX A Co‐infected NP swab samples among 1122 patients with positive multiplex PCR results, n = 23

**Table:**

Viruses	N = 23	%
Boca/P4	1	4.35
Cor 1/hMPV	1	4.35
Cor 1/Influenza A	2	8.70
Cor 1/Influenza B	3	13.04
Cor 1/RSV A	1	4.35
Cor 1/RSV B	1	4.35
Cor 2/Ent	1	4.35
Cor 2/Influenza A	2	8.70
Cor 2/Influenza B	1	4.35
Ent/Influenza A	1	4.35
hMPV/P2	1	4.35
Influenza A/Influenza B	1	4.35
Influenza A/Rhinov	1	4.35
Influenza A/RSV B	2	8.70
Influenza B/P1	1	4.35
Influenza B/P4	2	8.70
Rhinov/RSV B	1	4.35

*Note*: These 23 patients were excluded from the analysis due to co‐infection. Given the small sample size of 23/1122 (2%), we did not include co‐infections as a separate predictor variable in the regression models.

Abbreviations: Boca, bocavirus; hMPV, human metapneumovirus; RSV A/B, respiratory syncytial virus A/B; Rhinov, rhinovirus; Ent, Enterovirus; Cor 1/2, seasonal coronaviruses OC43, 229E/NL63; P1–P4, parainfluenza types 1–4.

## APPENDIX B Adjusted association between respiratory viral pathogens (vs. influenza) and need for mechanical ventilation and non‐invasive ventilation among hospitalized patients with COPD and a positive RV15 multiplex PCR test during winter seasons 2011–2015

**Table:**

Predictor	Odds ratio for mechanical ventilation (95% CI)
RSV infection vs. influenza infection	0.88 (0.47–1.67)
Non‐influenza, non‐RSV, viral infection vs. influenza infection	0.36 (0.19–1.68)
Age	0.98 (0.96–0.99)
BMI ≤ 18.5 kg/m^2^	0.84 (0.32–2.21)
Use of home oxygen	1.57 (0.88–2.80)
Clinical diagnosis of community acquired pneumonia	2.06 (1.34–3.16)
Current tobacco smoking	1.66 (1.04–2.66)

Predictor	Odds ratio for non‐invasive ventilation (95% CI)
RSV infection vs. influenza infection	3.05 (1.82–5.11)
Non‐influenza, non‐RSV, viral infection vs. influenza infection	1.62 (1.01–2.60)
Age	0.98 (0.96–1.00)
BMI ≤ 18.5 kg/m^2^	1.09 (0.50–2.37)
Use of home oxygen	2.56 (1.56–4.18)
Current tobacco smoking	2.03 (1.30–3.17)

*Note*: The reference variable is influenza infection (for RSV and other non‐influenza, non‐RSV respiratory viral infections). Variables including age, BMI, and use of home oxygen were forced into the model because of clinical significance. Clinical diagnosis of community acquired pneumonia, current smoking status, and admission from long‐term care were tested in the model and subjected to backwards selection methods.
